# Maternal Pre-Pregnancy Glycemic Status and Growth Delay in Korean Children Aged 18–36 Months: A Population-Based Study

**DOI:** 10.3390/jcm14207230

**Published:** 2025-10-14

**Authors:** Eun-Jung Oh, Yeeun Han, Tae-Eun Kim, Sang-Hyun Park, Hye Won Park, Hyuk Jung Kweon, Jaekyung Choi, Jinyoung Shin

**Affiliations:** 1 Department of Family Medicine, Konkuk University Medical Center, Chungju Hospital, Konkuk University School of Medicine, Chungju 27376, Republic of Korea; oej98@hanmail.net (E.-J.O.); fmkhj@kku.ac.kr (H.J.K.); 2 Department of Family Medicine, Konkuk University Medical Center, Konkuk University School of Medicine, Seoul 05030, Republic of Korea; 20240187@kuh.ac.kr (Y.H.); cjk@kuh.ac.kr (J.C.); 3 Department of Clinical Pharmacology, Konkuk University Medical Center, Seoul 05030, Republic of Korea; tekim@kuh.ac.kr (T.-E.K.); 20230583@kuh.ac.kr (S.-H.P.); 4 Department of Pediatrics, Konkuk University Medical Center, Konkuk University School of Medicine, Seoul 05030, Republic of Korea; 20110673@kuh.ac.kr

**Keywords:** glucose, prediabetes, diabetes mellitus, growth, administrative claims, health care

## Abstract

**Background/Objectives**: This study aimed at evaluating the association between maternal pre-pregnancy glycemic status and growth delay in offspring using nationwide health screening data. **Methods**: A retrospective cohort of 258,367 mother–child dyads born between 2014 and 2021 was analyzed. Maternal glycemic status was categorized as normal (<100 mg/dL), impaired fasting glucose (IFG, 100–125 mg/dL), or diabetes mellitus (DM, ≥126 mg/dL). Growth delay was defined as measurements below the 10th percentile of height, weight, and head circumference at 18–24 and 30–36 months. Visual and auditory development were assessed using caregiver questionnaires. Inverse probability of treatment weighting was applied, and weighted relative risks (RRs) were estimated. **Results**: The prevalence of growth delay was 3.5% for height, 3.8% for weight, and 4.3% for head circumference; visual and auditory problems were reported in 1.2% and 8.2% of children, respectively. Both the DM (1.2%) and IFG (9.3%) groups showed increased risks of growth delay across both age periods. Maternal hyperglycemia was also associated with offspring’s visual and auditory development, with age- and period-specific differences observed. **Conclusions**: Maternal pre-pregnancy glycemic status was significantly associated with delayed growth in Korean children aged 18–36 months. These findings highlight the importance of optimizing maternal glycemic control prior to pregnancy for favorable child developmental outcomes.

## 1. Introduction

Maternal diabetes mellitus (DM), including gestational DM (GDM) and pre-gestational DM, has been associated with an increased risk of pregnancy complications such as cesarean delivery [1,2]. It also contributes to adverse health outcomes in offspring, including growth delay, neonatal diseases, neurodevelopmental disorders, and metabolic disorders during childhood [3,4,5]. GDM, defined as glucose intolerance of varying severity first recognized during pregnancy, has shown a rising global prevalence, from 7.5% in 2009–2011 to 11.1% in 2012–2016 [6,7]. Evidence from a precious study suggests that GDM may account for approximately 18.6% of growth impairment in offspring, although this estimate may vary depending on the study population and methodology [8]. According to Korean national guidelines, all pregnant women are recommended to undergo an oral glucose tolerance test at 24–28 weeks of gestation, regardless of their underlying risk of GDM [9].

In the Taiwan Birth Cohort study involving 24,200 mother–child pairs, GDM was associated with a higher risk of obesity in offspring at 36 months than in children of mothers with normal blood glucose levels [10]. Elevated maternal blood glucose levels may promote fetal growth through maternal obesity, large for gestational age (LGA) status, and excessive gestational weight gain, although postnatal factors such as perinatal or infant care may also influence outcomes [8]. These effects are often more pronounced in cases of preexisting DM [11]. However, some studies have shown that these associations diminish with increasing age. For example, a Finnish study examining the combined effects of GDM and obesity found no significant association with child growth at birth or at 3, 6, 12, and 24 months [12]. Interestingly, positive correlations have been reported between maternal fat mass and children’s height and head circumference, suggesting a potential influence on early childhood development [12]. Despite these findings, few studies have examined the association between maternal pre-pregnancy glycemic status and offspring growth beyond birth weight, such as height and head circumference, or the impact of prediabetes on growth.

Emerging evidence suggests that maternal hyperglycemia may affect sensory development. For instance, a recent study from the Ma’anshan Birth Cohort (*n* = 1987) reported that higher maternal fasting glucose was associated with an increased risk of visual disorder in children aged 6 years [13]. A systematic review of seven studies suggested a possible link between GDM and newborn hearing acuity, although the evidence is heterogeneous and lacks long-term follow-up beyond 18 months [14].

In South Korea, the National Health Screening Program for Infants and Children (NHSPIC) initiated in 2007 aims at supporting normal growth and developmental milestones by monitoring and managing the growth of infants and children. Therefore, this study aimed at investigating the association between maternal pre-pregnancy glucose status and the risk of growth parameters (height, weight, and head circumference) and the underexplored domains of visual and auditory development using a large-scale, population-based cohort.

## 2. Materials and Methods

### 2.1. Study Data Sources and Participants

In this retrospective cohort study, we utilized data from the National Health Examination, jointly maintained by the National Health Insurance Service (NHIS) and NHSPIC. NHSPIC provides free-of-charge screening to all newborns nationwide, ensuring universal access regardless of socioeconomic status [15]. Growth and developmental assessments were conducted through surveys and physical examinations at seven intervals between 4 and 71 months of age (chronological age): 4–6, 9–12, 18–24, 30–36, 42–48, 54–60, and 61–71 months [16].

The NHIS conducts biennial health examinations for all individuals aged ≥ 20 years, collecting self-reported medical histories and anthropometric measurements. Its database contains comprehensive information on diagnoses, procedures, and prescriptions identified using International Classification of Diseases, Tenth Revision (ICD-10) codes [17].

**Figure 1:**
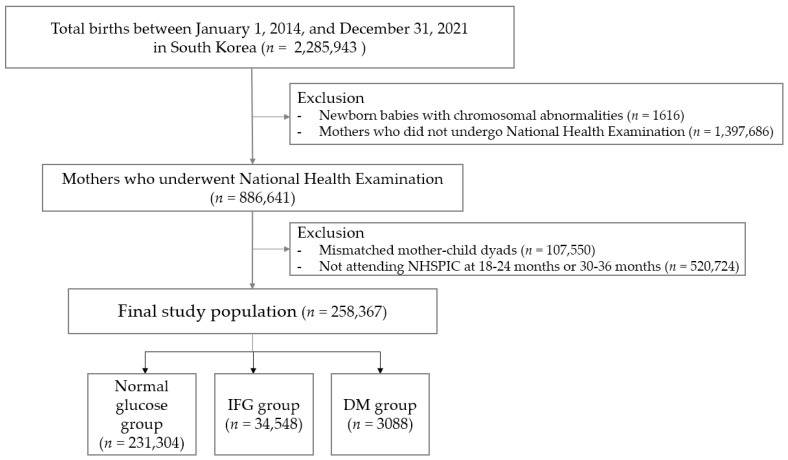
Study population.

Of the 2,285,943 births in South Korea between 1 January 2014 and 31 December 2021, we excluded 1616 cases with chromosomal abnormalities and 1,397,686 mothers who did not undergo health screening within three years before delivery, resulting in missing maternal information. The remaining 886,641 mothers were linked to their children’s records in the NHSPIC, using the family’s unique health insurance card number and delivery date. From this matched cohort of 779,091 pairs, we included 258,367 mother–child dyads who had completed both 18–24-month and 30–36-month NHSPIC examinations in the final analysis (Figure 1).

The study was conducted in accordance with the Declaration of Helsinki and approved by the Institutional Review Board (IRB) of the University Hospital (KUMC 2025-06-23, 12 June 2025). The requirement for informed consent was waived because this study did not include information on identifying individuals using previously collected data released from the general public.

### 2.2. Maternal Pre-Pregnancy Glycemic Status

Maternal glycemic status was categorized as normal glucose (<100 mg/dL), impaired fasting glucose (IFG: 100–125 mg/dL, and no prior prescription for antidiabetic medications), and DM (≥126 mg/dL, current use of antidiabetic medications, or diagnosis of DM based on ICD-10 codes O24.0–O24.3, E100–E109, E110–E119, E120–E129, E130–E139, and E140–E149). Classification was based on fasting glucose levels obtained from the NHIS health examination conducted within three years before delivery.

### 2.3. Growth Delay

Growth delay was defined as a height, weight, and head circumference below the 10th percentile. Although the World Health Organization (WHO) Growth Standards employ standard deviation scores for children under 36 months and percentiles for those aged ≥3 years, the NHSPIC adapted the use of percentile values for children under 3 years to reduce confusion stemming from differences in the interpretation of criteria [9]. In the NHSPIC, growth delay is typically defined as short stature and microcephaly below the 3rd percentile and underweight below the 5th percentile according to age-specific standards. In this study, we applied the 10th percentile threshold to enhance early identification of at-risk children. As children below this cutoff are subsequently referred for confirmatory diagnostic evaluation by a pediatric specialist [18], this approach is not expected to overestimate the prevalence of growth delay.

For children aged 18–24 months, the recumbent length was measured to the nearest 0.1 cm by two trained examiners using an infantometer, with the child in a supine position, head secured, and legs gently extended so that the heels contacted the footplate. Body weight was measured to the nearest 0.1 kg while the child was wearing light underwear without diapers. Head circumference was measured using a flexible, non-stretchable tape placed around the most prominent parts of the forehead and occiput with the child looking straight ahead. For children aged 30–36 months, standing height was measured without footwear, with the child standing upright in a proper posture while the headpiece was lowered to lightly compress the hair. Weight and head circumference were measured using the same methods as those used for the younger age groups.

### 2.4. Assessment of Visual and Auditory Development

Visual and auditory development were assessed using a caregiver-administered questionnaire without the objective diagnostic data (e.g., ophthalmological or audiological testing) (Supplementary Table S1). The questionnaire comprised four vision- and five auditory-related questions, with responses recorded as “Yes” or “No.” If an abnormal response was identified, the child was considered at risk of developmental impairment and was recommended for specialist evaluation. Sample items for children aged 18–24 months included the following: “ Does the child have difficulty making eye contact or exhibit nystagmus?” and “ Can the child say their own name (even if not perfectly articulated)?”. For children aged 30–36 months, sample items included the following: “ When one eye is covered, does the child seem to perceive a difference in vision between the eyes?” and “Does the child increase the TV volume louder than others?” [18].

### 2.5. Other Variables

Maternal BMI was calculated as the weight in kilograms divided by the square of height (kg/m^2^). Maternal comorbidities were identified using ICD-10 codes: pre-gestational hypertension (I10–I15, O10, prescription of antihypertensive medication, or systolic/diastolic blood pressure ≥ 140/90 mmHg), pregnancy-induced hypertension (ICD-10 codes O12–O15), GDM (O24.0), and depression (F329, F332, and F530). Preterm birth was defined as delivery before 37 weeks based on claims data. The delivery method (normal delivery and cesarean section) was identified using ICD-10 and procedural codes (O800–O809, O820–O829, R435, R436, RA314–318, RA36–38, RA43, R314, R450, R451, R4520, and R500). Child-related variables included gestational age at birth, sex, birth weight, and multiple births obtained from claims data. For multiple births, each child was treated as an independent observation and paired with the same maternal record according to pre-pregnancy glycemic status. As a result, the number of mother–child dyads equaled the number of offspring, although the same mother could contribute more than once. Major anomalies were also identified (Q00–Q98.4). Small for gestational age (SGA) was defined as birth weight below the 10th percentile (P050, P051, and P059). Neonatal intensive care unit (NICU) admission after birth was also identified (AJ101, AJ111, AJ121, AJ131, AJ144, AJ161, AJ201, AJ211, AJ221, AJ231, AJ244, AJ261, AJ301, AJ311, AJ321, AJ331, AJ351, and AJ051–AJ054).

### 2.6. Statistical Analysis

Continuous variables are presented as means ± standard deviations, and categorical variables are presented as numbers (%). Baseline characteristics across maternal BMI categories were compared using analysis of variance (ANOVA) for continuous variables and chi-square tests for categorical variables.

To adjust for baseline differences among maternal glucose categories, inverse probability of treatment weighting (IPTW) was applied based on propensity scores (PS) estimated using multinomial logistic regression (Supplementary Table S2). The covariates included maternal age, comorbidities, preterm birth, delivery method, gestational age, birth weight, sex, multiple births, major anomalies, SGA, and NICU admission after birth.

Weighted relative risks (RRs) and 95% confidence intervals (CIs) were calculated using generalized linear models with the normal glucose group serving as the reference category. The crude incidence rates (IRs) per 1000 individuals with developmental delays were also calculated. All statistical analyses were performed using the SAS software (version 9.4; SAS Institute Inc., Cary, NC, USA).

## 3. Results

### 3.1. Participant Characteristics by Maternal Glucose Status

Maternal pre-pregnancy glycemic status was classified into three groups: DM, IFG, and normal glucose (Table 1). Mothers with higher glucose levels were generally older, with higher BMI, and with a greater prevalence of pre-gestational hypertension, pregnancy-induced hypertension, gestational DM, and depression. In addition, the high-glucose group showed a higher proportion of preterm births and a lower proportion of vaginal deliveries.

The sex distribution of the offspring did not differ according to maternal glucose status. However, gestational age at birth tended to decrease as maternal glucose levels increased, although the mean gestational age remained above 32 weeks. The offspring of mothers with higher glucose levels had greater average birth weights, and both SGA and LGA infants were more frequent in the DM group. Multiple births were also more common among mothers with DM. Furthermore, the prevalence of major anomalies and NICU admissions after birth increased in parallel with the maternal glucose levels.

### 3.2. Growth Delay, Visual, and Auditory Problems of Offspring

As shown in Table 2, the risk of growth delays in height, weight, and head circumference at both 18–24 and 30–36 months increased proportionally with maternal glucose levels. Compared to the normal glucose group, the IFG group had a significantly higher risk of growth delays across all three parameters (height, weight, and head circumference).

Incidence rates were calculated per 1000 people. In the IFG group, the increase in RR for visual developmental problems at 18–24 months was not statistically significant compared to the normal glucose group, whereas in the DM group, the risk was significantly elevated. Among children aged 30–36 months, both the IFG and DM groups exhibited a higher risk of visual developmental problems than the normal group. In the auditory assessment, the IR of auditory developmental problems was consistently higher in the IFG and DM groups than in the normal glucose group at both time points. However, a significant increase in the RR was observed only in the IFG group, whereas the DM group exhibited a paradoxically lower risk.

## 4. Discussion

This study investigated the association between maternal pre-pregnancy glycemic status and growth delays in children within the first three years of life, using a large, population-based cohort. Both the IFG and DM groups were associated with an increased risk of growth delays in height, weight, and head circumference compared with the normal glucose group. The risk of growth delays increased proportionally with higher maternal glucose levels, and this pattern was consistently observed in both the 18–24-month and 30–36-month assessments.

Regarding visual development, the risk of visual problems was elevated in the DM group at 18–24 months, whereas at 30–36 months, increased risks were observed in both the IFG and DM groups. These findings suggest that the risk of visual problems tends to increase as children grow older. For auditory function, a higher risk was noted in the IFG group, and although the incidence decreased at 30–36 months, a significant association persisted.

Previous studies have reported that elevated maternal glucose levels, often driven by obesity and overnutrition, increase the risk of childhood obesity [19]. However, our findings underscore that even pre-pregnancy elevations in glucose, particularly in the prediabetic range, are associated not only with impaired physical growth but also with underdevelopment of visual and auditory functions. This suggests that subtle disturbances in maternal glucose homeostasis may have substantial consequences for offspring development.

Several mechanisms may explain this association. Maternal hyperglycemia can induce metabolic alterations, including greater glycemic variability, dysregulated hormone secretion (e.g., adipokines and leptin), and chronic low-grade inflammation [12]. Additionally, maternal obesity is linked to shorter gestational periods, which may contribute to postnatal growth delays [3]. In our study, even after adjusting for gestational age, the DM group still exhibited a reduced gestational duration, potentially contributing to growth impairment. While we did not directly assess catch-up growth, previous research indicates that rapid catch-up growth occurs during the first year of life, followed by slower, plateaued growth in early childhood, with gestational age being a key determinant [20]. Consistent with our findings, a recent study of 855 GDM mothers reported increased rates of LGA, SGA, preterm birth, and fetal distress [21]. Therefore, growth outcomes should be evaluated in the context of pre-pregnancy weight status, gestational weight gain, and GDM diagnosis [22].

Potential mechanisms underlying growth delays include short-term effects, such as fetal neuroinflammation and iron deficiency, and long-term effects, such as epigenetic alterations, dysregulated lipid metabolism, and structural developmental anomalies [23]. Hyperglycemia may also impair visual development via abnormal retinal maturation, delayed differentiation of retinal cells, and elevated oxidative stress [24].

Interestingly, the incidence of auditory problems was lower in the DM group than in the normal glucose group. Evidence linking maternal hyperglycemia to auditory development in offspring is limited and inconsistent. The prevalence of permanent hearing impairment in healthy newborns ranges from 0.1% to 0.3% [25]. In a Chinese newborn hearing screening study involving 369 infants, hearing loss was detected in 5.69% of those born to mothers with GDM compared to 1.22% in the non-GDM group (*p* = 0.031). The biological mechanisms underlying these associations are not fully understood. Proposed pathways include oxidative stress, excessive free radical production leading to cellular damage, impaired organogenesis, and altered insulin-like growth factor-1 (IGF-1) levels, which may affect cochlear morphogenesis [26,27]. Microvascular injury during pregnancy-induced hyperglycemia could also lead to ischemia in the inner ear; however, recovery in the early postnatal period or early interventions may have mitigated such effects, resulting in a lower observed incidence at the time of assessment [28]. Another possible explanation is that mothers with DM generally receive more intensive prenatal and postnatal care. In South Korea, initial newborn hearing screening is performed within one day of birth for 96.5% of infants and within three days for 97% [29]. Newborns of mothers with DM are more likely to be admitted to the NICU or to undergo enhanced observation immediately after birth [30]. Such increased surveillance may facilitate earlier detection and intervention for auditory problems, potentially mitigating minor deficits before formal assessment and thereby contributing to the lower observed incidence of auditory impairment.

Although the relative risks observed in this study were modest, consistent risks should not be underestimated in a population-based context. Because maternal hyperglycemia is highly prevalent, even minor relative increases in adverse child outcomes could result in substantial public health implications. These findings highlight the need for continued attention to maternal metabolic health and systematic child monitoring strategies.

This study has several limitations. First, maternal glycemic status was determined from registry data and measured only once within three years before delivery, without precise timing, making it susceptible to temporary fluctuations. The BMI values were also measured at the same time as pre-pregnancy glucose and were available only once. Therefore, they may not reflect maternal weight changes during pregnancy or after delivery. Because repeated measurements were not available in our dataset, further studies with longitudinal assessments are needed to define maternal glycemic status and BMI. Second, socioeconomic status, educational level, smoking, alcohol consumption, and physical activity were unavailable in our dataset and thus could not be included as covariates in the analyses. These covariates, which strongly influence nutritional status or access to health care, particularly in lower-income groups, may have an effect on the growth status. Third, visual and auditory problems were identified through caregiver questionnaires, though referrals for specialist evaluation were made later. Our findings of visual and auditory problems do not represent clinically confirmed diagnoses. Finally, although growth delays in height, weight, head circumference, and sensory development were observed, we could not account for catch-up growth, which can be nonlinear during early childhood and is influenced by genetic and environmental factors [20,31].

## 5. Conclusions

In this large, population-based cohort study, higher maternal pre-pregnancy glucose levels, particularly in the IFG and DM ranges, were associated with an increased risk of growth delays in height, weight, and head circumference during early childhood. Maternal hyperglycemia was also associated with alterations in visual and auditory development, although these effects varied with age and the assessment period. These findings highlight the importance of optimizing maternal glycemic control prior to conception to support healthy fetal and early childhood development. Early life interventions—particularly dietary and lifestyle modifications—may mitigate the adverse effects of maternal hyperglycemia. Future research should further investigate the underlying mechanisms by which maternal glycemic status influences offspring growth and development, taking into account genetic and environmental factors as well as potential long-term effects. Identifying these mechanisms could inform targeted interventions to improve maternal and child health outcomes.

## Figures and Tables

**Table 1:** Baseline characteristics of the study population (*n* = 258,367).

Variables	Normal Glucose	IFG	DM	*p*-Value
Mother–child dyads, *N* (%)	231,304 (89.5)	23,975 (9.3)	3088 (1.2)	
Maternal Characteristics				
Maternal age, years	32.24 ± 3.9	32.96 ± 4.1	34.17 ± 4.3	<0.001
Maternal age, ≥35 years	60,544 (26.2)	8028 (33.5)	1398 (45.3)	<0.001
Body mass index, kg/m^2^	21.61 ± 3.2	22.82 ± 3.9	25.68 ± 5.2	<0.001
Pre-gestational hypertension	4506 (2.0)	723 (3.0)	345 (11.2)	<0.001
Pregnancy-induced hypertension	24,764 (10.7)	3129 (13.1)	604 (19.6)	<0.001
Gestational DM	33,457 (14.5)	5495 (22.9)	1666 (54.0)	<0.001
Depression	4028 (1.7)	461 (1.9)	89 (2.9)	<0.001
Preterm birth	9728 (4.2)	1172 (4.9)	277 (9.0)	<0.001
Vaginal delivery	129,697 (56.1)	12,124 (50.6)	1059 (34.3)	<0.001
Offspring Characteristics				
Sex, male	118,058 (51.0)	12,202 (50.9)	1583 (51.3)	0.882
Gestational age at birth, weeks	35.43 ± 2.4	35.17 ± 2.5	34.85 ± 2.4	<0.001
Birthweight, mean, kg	3.18 ± 0.4	3.22 ± 0.5	3.26 ± 0.6	<0.001
Birthweight group				<0.001
<2 kg	2354 (1.0)	277 (1.2)	60 (1.9)	
2–3 kg	60,480 (26.2)	5736 (23.9)	771 (25.0)	
3–4 kg	161,695 (69.9)	16,807 (70.1)	1897 (61.7)	
≥4 kg	6775 (2.9)	1155 (4.8)	360 (11.7)	
Multiple birth	8220 (3.6)	938 (3.9)	183 (5.9)	<0.001
Major anomaly	14,611 (6.3)	1616 (6.7)	357 (11.6)	<0.001
Small for gestational age	1953 (0.8)	162 (0.7)	37 (1.2)	<0.001
NICU admission	13,769 (6.0)	1695 (7.1)	497 (16.1)	<0.001

Data are presented as number (%) or mean ± standard deviation. IFG: impaired fasting glucose; DM: diabetes mellitus; NICU: neonatal intensive care unit.

**Table 2:** The risks of growth, visual, and auditory problems of offspring according to maternal glycemic status.

		18–24 Months				30–36 Months			
Glycemic Status	*N*	Event	IR	RR (95% CI)	*p*-Value	Event	IR	RR (95% CI)	*p*-Value
Height < 10 Percentile									
Normal	231,304	8104	3.50	1 (ref.)		9434	4.08	1 (ref.)	
IFG	23,975	862	3.60	1.064 (1.033–1.097)	<0.001	979	4.08	1.036 (1.008–1.065)	<0.001
DM	3088	180	5.83	1.150 (1.117–1.185)	<0.001	168	5.44	1.039 (1.010–1.069)	<0.001
Weight < 10 Percentile									
Normal	231,304	8897	3.85	1 (ref.)		8975	3.88	1 (ref.)	
IFG	23,975	874	3.65	1.078 (1.047–1.109)	<0.001	882	3.68	1.077 (1.047–1.108)	<0.001
DM	3088	148	4.79	1.646 (1.603–1.690)	<0.001	134	4.34	1.354 (1.318–1.391)	<0.001
Head Circumference < 10 Percentile									
Normal	231,304	9793	4.23	1 (ref.)		10,934	4.73	1 (ref.)	
IFG	23,975	1090	4.55	1.143 (1.113–1.174)	<0.001	1188	4.96	1.128 (1.100–1.157)	<0.001
DM	3088	192	6.22	1.234 (1.202–1.267)	0.073	195	6.31	1.406 (1.373–1.441)	<0.001
Visual Developmental Problem									
Normal	231,304	2689	1.16	1 (ref.)		3748	1.62	1 ( ref.)	
IFG	23,975	303	1.26	1.019 (0.966–1.074)	0.496	437	1.82	1.103 (1.056–1.152)	<0.001
DM	3088	57	1.85	1.236 (1.174–1.301)	<0.001	69	2.23	1.313 (1.259–1.370)	<0.001
Auditory Developmental Problem									
Normal	231,304	18,598	8.04	1 (ref.)		5505	2.38	1 (ref.)	
IFG	23,975	2168	9.04	1.101 (1.08–1.122)	<0.001	681	2.84	1.154 (1.113–1.196)	<0.001
DM	3088	307	9.94	0.952 (0.933–0.971)	<0.001	118	3.82	0.823 (0.791–0.855)	<0.001

IFG: Impaired Fasting Glucose; DM: Diabetes Mellitus; RR: Relative Risk; CI: Confidence Interval.

## Data Availability

The data supporting this article are accessible from the National Health Insurance Service Open Data Portal (https://nhiss.nhis.or.kr/, accessed on 18 June 2025).

## Supplementary Materials

The following supporting information can be downloaded at: https://www.mdpi.com/article/10.3390/jcm14207230/s1. Table S1: Questionnaire for visual and auditory development in NHSPIC, Table S2: Baseline Characteristics of the Study Population Before and After IPTW.

## Abbreviations

The following abbreviations are used in this manuscript:

BMI	body mass index
CIs	confidence intervals
DM	diabetes mellitus
GDM	gestational diabetes mellitus
ICD-10	International Classification of Diseases, Tenth Revision
IFG	impaired fasting glucose
IRs	incidence rates
IRB	Institutional Review Board
IPTW	inverse probability of treatment weighting
LGA	large for gestational age
NHIS	National Health Insurance Service
NHSPIC	National Health Screening Program for Infants and Children
NICU	neonatal intensive care unit
PS	propensity scores
RRs	relative risks
SGA	small for gestational age
