# Supplementary material for: Maternal Pre-Pregnancy Glycemic Status and Growth Delay in Korean Children Aged 18–36 Months: A Population-Based Study
Source: J Clin Med. 2025 Oct 14;14(20):7230. doi: 10.3390/jcm14207230 (PMC12564939; doi:10.3390/jcm14207230)
Supplement: Supplementary file 1 [file jcm-14-07230-s001.zip › jcm-3859190-supplementary.pdf]

Supplementary Table S1. Questionnaire for visual and auditory development in NHSPIC

	18-24 months	30-36 months
Visual 1	Does the child have difficulty making eye contact or exhibit nystagmus?	Does the child have difficulty making eye contact or exhibit nystagmus?
Visual 2	Does the pupil appear cloudy?	When looking at objects in front, does the child frequently turn their face sideways or tilt their head?
Visual 3	When looking at objects in front, does the child frequently turn their face sideways or tilt their head?	Does the child view book, television, or objects at an excessively close distance or squint while looking at them?
Visual 4	Does the child view book, television, or objects at an excessively close distance or squint while looking at them?	When one eye is covered, does the child seem to perceive a difference in vision between the eyes?
Auditory 1	Can the child distinguish sounds of normal volume from all directions?	Is the number of words the child speaks steadily increasing?
Auditory 2	Does the child understand and respond to simple yes/no questions (e.g., "Are you hungry?" "Do you need to urinate?")?	Can the child combine two words to form simple phrases (e.g., "Give me all," "Read the book")?
Auditory 3	Can the child say their own name (even if not perfectly articulated)?	Does the child increase the TV volume louder than others?
Auditory 4	When asked to identify a picture in a book, can the child point to the correct one?	Can the child pronounce words containing consonants such as /k/, /t/, /p/, or /g/?
Auditory 5	Does the child understand and follow simple verbal instructions (e.g., "Give me the cup," "Bring the ball")?	Has the child experienced recurrent episodes of acute otitis media (≥4 times in 6 months or ≥6 times in 1 year)?

Supplementary Table S2. Baseline Characteristics of the Study Population Before and After IPTW

**Characteristics**	**Normal glucose**	**IFG**	**DM**	**ASD**	**Normal glucose**	**IFG**	**DM**	**ASD**
Mother-child dyads	231,304 (89.5)	23,975 (9.3)	3088 (1.2)		231,304 (89.5)	23,975 (9.3)	3088 (1.2)	
			Maternal characteristics					
Maternal age, years	32.24±3.9	32.96±4.1	34.17±4.3	0.1776	32.36±3.93	32.27±12.47	32.15±33.8	0.0106
Maternal age, ≥ 35 years	60,544 (26.2)	8028 (33.5)	1398 (45.3)		64,195.9 (27.5)	61,912.3 (27.3)	60,043.5 (26.9)	
Body mass index, kg/m²	21.61±3.2	22.82±3.9	25.68±5.2	0.3409	22.2±6.18	21.74±10.39	21.44±29.67	0.0539
Pregestational hypertension	4506 (2.0)	723 (3.0)	345 (11.2)	0.3218	5030.2(2.2)	4816.6(2.1)	4773.5(2.1)	0.0023
Pregnancy-induced hypertension	24,764 (10.7)	3129 (13.1)	604 (19.6)	0.1769	25,596.3(10.96)	24,833.3(10.93)	24,158.8(10.82)	0.0035
Gestational DM	33,457(14.5)	5495(22.9)	1666(54.0)	0.6731	33,965.5(14.54)	47,364.3(20.85)	99,153.7(44.42)	0.5194
Depression	4028 (1.7)	461 (1.9)	89 (2.9)	0.0627	4103.3(1.76)	4043.3(1.78)	4540.3(2.03)	0.0186
Preterm birth	9728 (4.2)	1172 (4.9)	277 (9.0)	0.1616	10,052.4(4.3)	9889.3(4.35)	9028.4(4.04)	0.0154
Vaginal delivery	129,697 (56.1)	12,124 (50.6)	1059 (34.3)	0.3339	129,821.4(55.59)	125,689(55.32)	131,587.9(58.95)	0.0733
			Offspring characteristics					
Sex, male	118,058 (51.0)	12,202 (50.9)	1583 (51.3)	0.0074	118,370.3(50.69)	116,083.8(51.09)	110,523.2(49.51)	0.0316
Gestational age, weeks	35.43±2.4	35.17±2.5	34.85±2.4	0.1076	35.42±2.39	35.16±7.55	34.92±15.14	0.0462
Birthweight, mean, kg	3.18±0.4	3.22±0.5	3.26±0.6	0.0953				
Birthweight group				<0.001				0.0022
<2 kg	2354 (1.0)	277 (1.2)	60 (1.9)		2403.7(1.03)	2513.5(1.11)	2545.5(1.14)	
2–3 kg	60,480 (26.2)	5736 (23.9)	771 (25.0)		59,910.5(25.65)	60,330.1(26.55)	79,468.0(35.6)	
3–4 kg	161,695 (69.9)	16,807 (70.1)	1897 (61.7)		164,007.1(70.23)	156,389.8(68.84)	132,793.4(59.49)	
≥ 4 kg	6775 (2.9)	1155 (4.8)	360 (11.7)		7204.1(3.08)	7961.7(3.5)	8415.5(3.77)	
Multiple birth	8220 (3.6)	938 (3.9)	183 (5.9)	0.0932	8400.2(3.6)	8291.2(3.65)	8088.2(3.62)	0.0028
Major anomaly, N (%)	14,611 (6.3)	1616 (6.7)	357 (11.6)	0.1678	14,893.1(6.38)	14,583.7(6.42)	11,932(5.35)	0.0456
SGA, N (%)	1953 (0.8)	162 (0.7)	37 (1.2)	0.0543	1934.5(0.83)	1808.7(0.8)	2918.8(1.31)	0.0502

NICU admission, n (%)	13,769 (6.0)	1695 (7.1)	497 (16.1)	0.2849	14,335.6(6.14)	13,914.2(6.12)	12,280.5(5.5)	0.0266

Data was shown as N (%) or mean value ± standard deviation.

IPTW: Inverse probability of treatment weighting, ASD: Absolute Standardized Difference, IFG: Impaired fasting glucose, DM: Diabetes Mellitus, SGA: Small for Gestational Age, NICU: Neonatal Intensive Care Unit
